# Effect of enzymatic modification on the structure and rheological properties of diluted alkali-soluble pectin fraction rich in RG-I

**DOI:** 10.1038/s41598-024-62180-2

**Published:** 2024-05-20

**Authors:** Adrianna Kaczmarska, Piotr M. Pieczywek, Justyna Cybulska, Artur Zdunek

**Affiliations:** Institute of Agrophysics, Polish Academy of Sciences, Doświadczalna 4, 20-270 Lublin, Poland

**Keywords:** Carbohydrates, Enzymes, Cell wall, Dietary carbohydrates, Polysaccharides

## Abstract

This study focuses on pectin covalently linked in cell walls from two sources, apples and carrots, that was extracted using diluted alkali, and it describes changes in the rheological properties of diluted alkali-soluble pectin (DASP) due to enzymatic treatment. Given DASP’s richness of rhamnogalacturonan I (RG-I), RG-I acetyl esterase (RGAE), rhamnogalacturonan endolyase (RGL), and arabinofuranosidase (ABF) were employed in various combinations for targeted degradation of RG-I pectin chains. Enzymatic degradations were followed by structural studies of pectin molecules using atomic force microscopy (AFM) as well as measurements of rheological and spectral properties. AFM imaging revealed a significant increase in the length of branched molecules after incubation with ABF, suggesting that arabinose side chains limit RG-I aggregation. Structural modifications were confirmed by changes in the intensity of bands in the pectin fingerprint and anomeric region on Fourier transform infrared spectra. ABF treatment led to a decrease in the stability of pectic gels, while the simultaneous use of ABF, RGAE, and RGL enzymes did not increase the degree of aggregation compared to the control sample. These findings suggest that the association of pectin chains within the DASP fraction may rely significantly on intermolecular interactions. Two mechanisms are proposed, which involve side chains as short-range attachment points or an extended linear homogalacturonan conformation favoring inter-chain interactions over self-association.

## Introduction

Pectin constitutes up to 35% of plant cell walls, performs important functions in plant growth and development, maintains cell–cell integrity, and, in the case of fruit and vegetables, determines firmness and texture^1,2^. Pectin is considered to be the component providing visco-plastic properties to the load-bearing cellulose–hemicellulose network in the cell wall; therefore, it plays an important role in plant cell wall rheology^3^. Pectin is also important for the food industry due to its ability to increase viscosity and bind water. Moreover, pectin gelling activity may be tuned by various parameters, such as the structure and concentration of pectin, pH, temperature, and presence of cations^4,5^.

There are three main pectic domains. Homogalacturonan (HG) consists of a linear chain of α-(1,4)-linked D-galacturonic acid (GalA) and is known as pectin’s “smooth” region. Rhamnogalacturonans belong to the so-called “hairy” region. The backbone of rhamnogalacturonan I (RG-I) is made of the diglycosyl repeating unit [→ 4-α-d-GalpA-(1 → 2)-α-l-Rhap-(1 →]. Predominantly, a large proportion of rhamnose units are substituted at O-4 with side chains composed principally of arabinans, galactans, and/or arabinogalactans^1^.

Neutral side chain loss and the rearrangement of their associations within RG-I are some of the most pronounced and earliest changes in pectin structure during maturation, ripening, and storage in fruits and vegetables. The majority of structural changes are associated with β-galactosidase and α-L-arabinofuranosidase (ABF) enzymatic activity, which is observed during ripening, especially for firmly bound polymers extracted by sodium carbonate^6^. The percentage of methyl-esterified GalA units within the HG substructure is defined as the degree of methyl esterification (DM), while the percentage of O-acetylated GalA units is the degree of acetylation (DA). The numbers of methyl and acetyl groups in pectin chains affect the gelling conditions and the viscosity of pectin solutions; thus, they are some of the major factors determining the functionality of pectin chains. The DM and DA are strongly influenced by the plant source as well as the extraction method. For low-methylated (LM) pectin (DM < 50%), gelation occurs at acidic pH (2–6) and in the presence of divalent ions such as Ca^2+^, while high-methylated (HM) pectin (DM > 50%) form gels in the presence of greater than 55% sugar or a similar co-solute at pH < 3.5^7^. Hydrogen bonds and electrostatic interactions play a crucial role in the gelation mechanism of HM pectin, while for LM pectin the “egg-box” model describes binding processes and junction zone formation between non-esterified GalA units and calcium ions^8^. Characteristics of the pectin polymer backbone, including its intrinsic flexibility or stiffness, play a major role in the rheological properties in solution and influence the order/disorder state of the system on a supramolecular scale, especially while different levels of chain association may be involved in network formation^9^.

The diluted alkali-soluble pectin (DASP) fraction of the cell wall pectic matrix extracted with sodium carbonate is considered to be the covalently linked fraction of the cell wall. As previously reported, these molecules show the distinctive feature of creating a self-assembled network on mica^10^. Atomic force microscopy (AFM) imaging and coarse grain simulations have confirmed that the network-like appearance on mica originates from rhamnose units separating two sections of HG and the creation of kinks at the characteristic angle of 118°^11^. Previous studies have demonstrated that DASP from fruits like pear or apple shows gelling ability dependent on concentration, pH, or monovalent and divalent cations in aqueous medium, indicating the possible application of this polysaccharide fraction due to its low methylation level^12^. However, due to the significantly different conformations of RG-I and HG^2^, we hypothesize that the rheology of DASP, which is rich in RG-I^13^, may be largely affected by the side chains and the presence of the kinks caused by rhamnose interspaced with GalA.

The goal of this paper was structural characterization at the supramolecular scale and investigation rheological properties of the DASP fractions extracted from two horticultural sources (apple and carrot) and structurally changed by enzymatic modification. In this experiment, RG-I acetyl esterase (RGAE), rhamnogalacturonan endolyase (RGL), and ABF were used. RGAE is an enzyme that participates in the deacetylation of GalA in the RG-I backbone. RGL participates in the endotype eliminative cleavage of L-α-rhamnopyranosyl-1,4-α-D-galactopyranosyluronic acid bonds of RG-I domains in ramified hairy regions of pectin, leaving L-rhamnopyranose at the reducing end and 4-deoxy-4,5-unsaturated D-galactopyranosyluronic acid at the non-reducing end^14,15^. ABF preferentially removes α-1,2- and α-1,3-linked arabinose from side chains of either arabinan or arabinoxylan, and it hydrolyses α-1,5-linked arabino-oligosaccharides at a low rate^14^. It is hypothesized that the detachment of the rhamnose and side chains affects pectin rheology. In this study, the effect of selective modification of pectin chains was compared for apple and carrot DASP and studied by high-performance liquid chromatography (HPLC), Fourier transform infrared (FTIR) spectroscopy, AFM imaging analysis, and rheological measurements of pectin aqueous solution.

## Results and discussion

### Nanostructure

**Figure 1:**
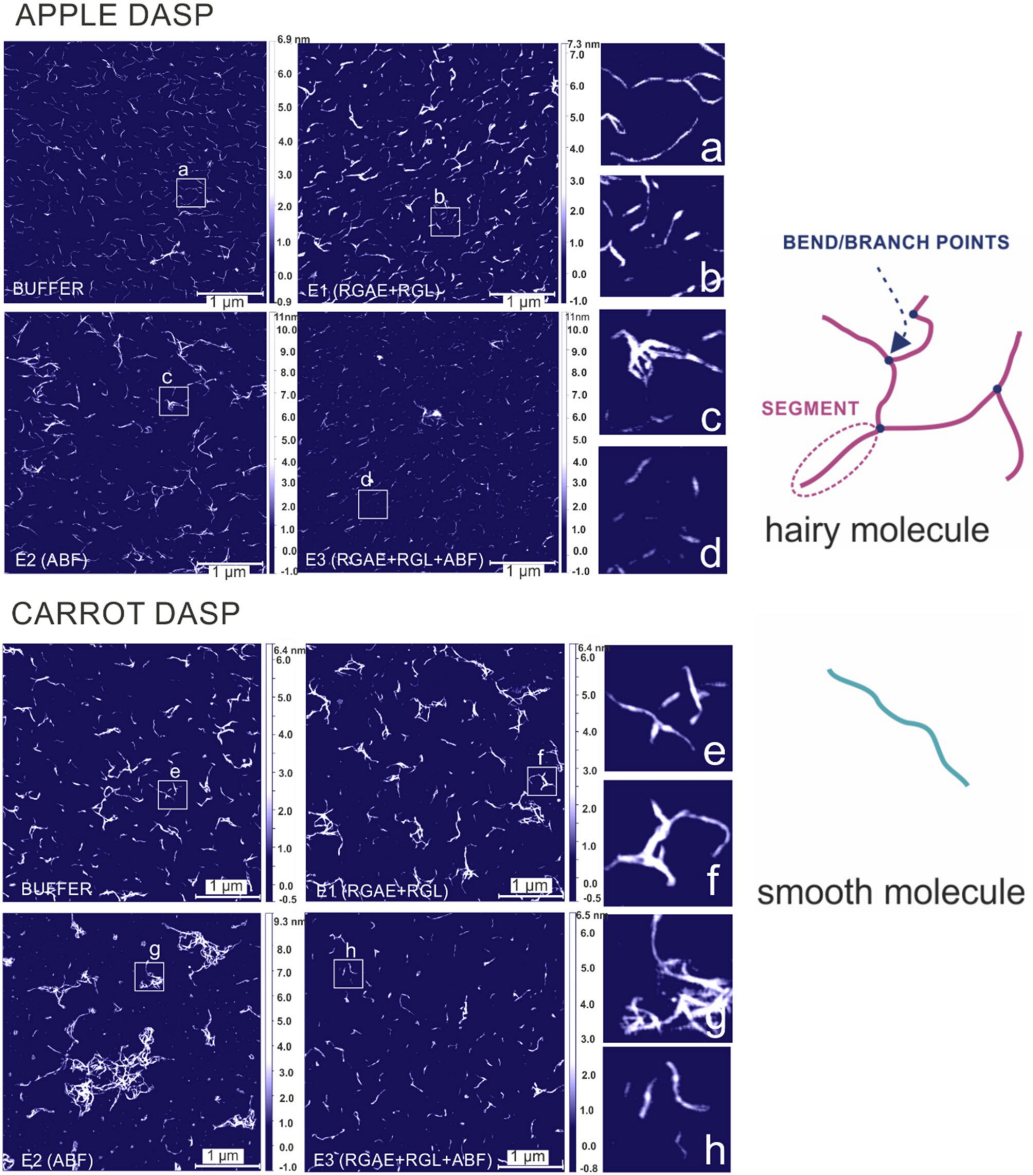
AFM height images of control (buffer) and enzymatically modified (incubation time 120 min) apple DASP (**a**–**d**) and carrot DASP (**e**–**h**) on mica. Scale bar represents 1 µm. Illustrations show two main categories of structures detected on topological AFM images: hairy and smooth molecules.

Figure 1 shows representative AFM height images of DASP from apple and carrot in the control (buffer) batch and after 120 min of incubation in three enzymatic cocktails: E1 (RGL + RGAE), E2 (ABF), and E3 (RGL + RGAE + ABF). Zoomed regions of the DASP incubated in the buffer presented in Fig. 1a,f show fibers only in the form of rod-like structures with linear segments of variable length and separated by bending or branching points. Such features have been previously observed for DASP fractions extracted from apple, carrot, or pear^10,16^ and have been explained as the result of rhamnose interspersion with GalA^11^. A similar structure was created on the mica by the DASP fibers after applying the E1 treatment (Fig. 1b,g). In the sample treated with E2 (Fig. 1c,h), larger aggregates and longer chains were observed and were particularly pronounced for carrot (Fig. 1h). Additionally, with both the E2 treatment and the combination of the three enzymes (E3) (Fig. 1d,i), short chains and small molecules were mostly noted.

**Figure 2:**
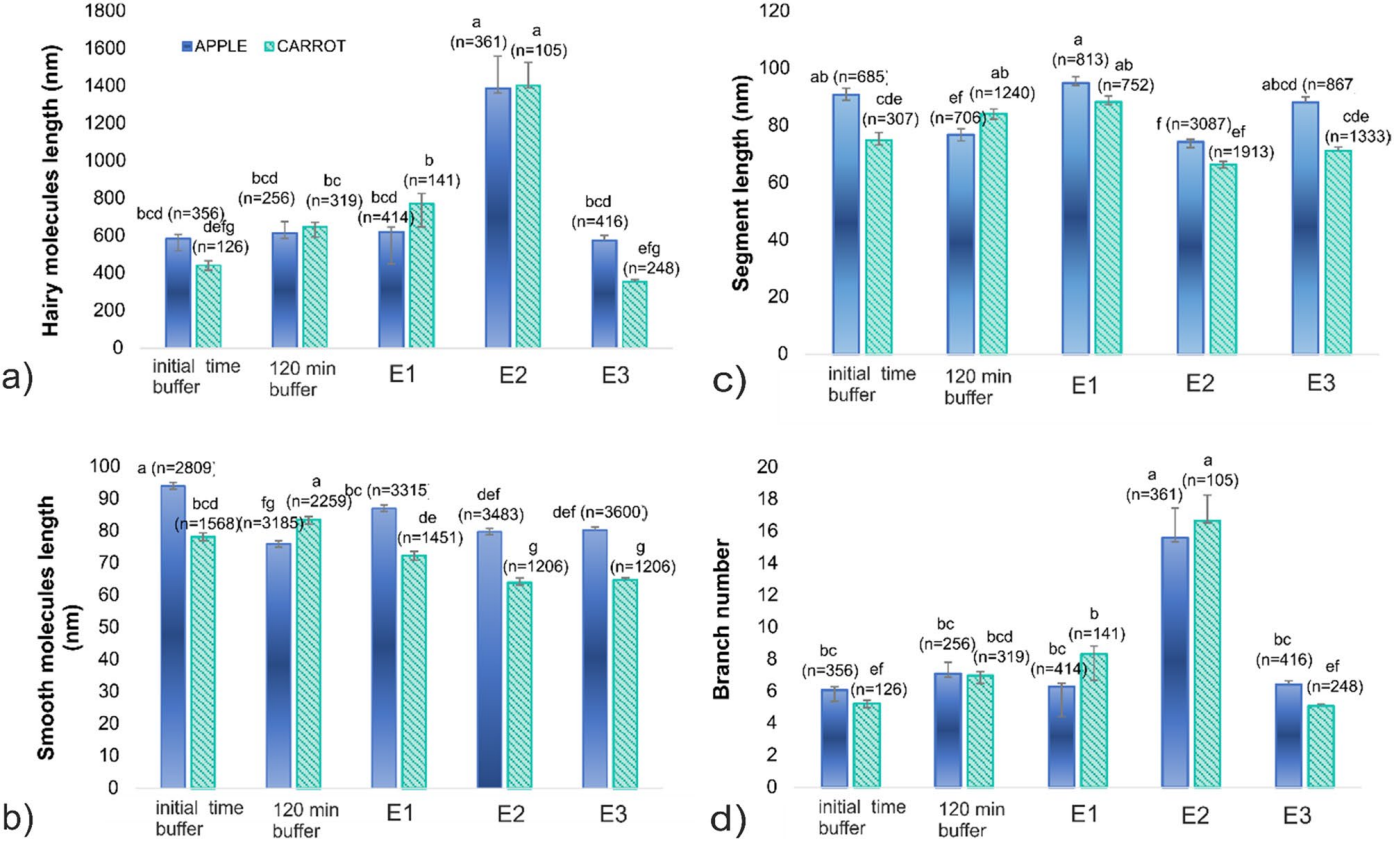
Changes in structural parameters of DASP molecules from apple and carrot treated with various combinations of enzymatic modification after 120 min. E1: DASP water solution with RGAE and RGL; E2: DASP water solution with ABF; E3: DASP water solution with RGAE, RGL, and ABF. Bars represent standard error (*n* is the number of replicates). Different letters indicate statistically significant differences (ANOVA, *p* < 0.05).

According to the image analysis performed on the AFM scans, the observed structures were categorized as “hairy” molecules or “smooth” molecules. Additionally, the total length of the branched molecule was calculated as the sum of lengths of the branches belonging to the molecule. The average total lengths of the molecules classified as hairy, before being placed in buffer, were approximately 585 ± 23 nm and 443 ± 23 nm for apple DASP (DASP-A) and carrot (DASP-C), respectively (Fig. 2a). This length is consistent with that previously obtained by another study^17^, which was from 20 to 1000 nm for the sodium carbonate pectin fraction extracted from different fruits. Contour lengths of alkali-treated sugar beet pectin were in the range of 20–520 nm^18^, similar to the molecule length for Na_2_CO_3_ extracts obtained from mature green tomato fruits (20–400 nm)^19^.

After placing the pectin in the buffer, the lengths of hairy molecules (Fig. 2a) did not change significantly for apple, while they increased for carrot. The length of molecules classified as smooth, before being placed in buffer (Fig. 2b), was shorter than 100 nm, and the incubation with buffer for 120 min caused apparent shortening for apple and a slight increase for carrot (Fig. 2b). The effect of incubation with buffer, reflected by changes in parameters after 120 min of incubation, suggests that pectin incubated in buffer alone may undergo structural changes leading to self-aggregation. Since pectin was incubated in buffer at pH 7, this result may be similar to that previously obtained for DASP-A and may be explained by the mechanism of high electrostatic repulsion between fully dissociated macromolecules that probably blocked the formation of extended pectin chains^4^.

Enzymatic treatment with cocktails E1 and E3 did not cause a statistically significant change in the length of the hairy molecules extracted from apple (Fig. 2a). In the case of carrot (Fig. 2a), treatment with E1 caused a slight but non-significant increase in length, while treatment in E3 caused a statistically significant decrease compared to incubation with buffer. Analysis of the number of segments (Fig. 2d) and the average length of segments (Fig. 2c) revealed that the total length of hairy molecules was related to the number of segments but that, simultaneously, the segments seemed to become slightly shorter after incubation with enzymes. The most pronounced effect on the structure of DASP in both materials was obtained when E2 treatment was applied (Fig. 2a). ABF was the only active enzyme in the E2 treatment and caused a significant increase in the total length of hairy molecules (up to almost 1.5 µm after 120 min). This effect was clearly associated with an increase in the number of segments and their only slight shortening during incubation (Fig. 2d). It is also worth noting that the chains classified as smooth (Fig. 2b) had lengths similar to those of segments of hairy molecules (Fig. 2b), i.e., less than 100 nm. The smooth molecules were unaffected by enzymatic treatment even with E2 (Fig. 2b). As ABF preferentially removes α-1,2- and α-1,3-linked arabinose from side chains, the effect of E2 on the structure of pectin molecules could be explained by the gradual removal of arabinose, followed by aggregation of RG-I molecules, thereby resulting in a three-fold increase in the number of branches per molecule (from about five to 15 segments per molecule), as shown in Fig. 2d.

Contrary to the application of ABF alone, the simultaneous action of ABF with RGAE and RGL enzymes (E3 treatment) did not result in aggregation and did not increase the lengths of hairy molecules. The lengths of hairy molecules after incubation in E3 were 576 ± 26 nm and 356 ± 12 nm for DASP-A and DASP-C, respectively. A decrease in the number of branches per molecule, from seven side branches for both sources to six for apple pectin and five for carrot pectin, was also observed for this treatment. Shortening of hairy molecules and a decrease in the number of branches due to E3 were more pronounced for DASP-C than for DASP-A. This higher fragmentation of the carrot pectin chain may have resulted from the greater RG-I content (62.90 mol%) than in apple (41.50 mol%), as was previously described^13^, which provides more sites of action for pectinolytic enzymes.

Incubation in the E1 enzyme mixture did not cause significant differences in branch lengths (Fig. 2c) for either source. It is suspected that the function of the RGL enzyme may have been impaired because the abundance of arabinose side chains prevented access to the chain due to steric hindrance.

A slight decrease in segment length (Fig. 2c), which could be attributed to the lengths of side branches, was noted for the E2 treatment. Moreover, for the combination of the three enzymes (E3), a decrease in the average length of smooth molecules (Fig. 2b) was also observed, indicating chain fragmentation. This may suggest that the simultaneous action of the enzymes modifying the RG-I skeleton and the enzymes that remove the arabinose side chains allowed for more effective fragmentation of the pectin chains. Hence, this result supports the above explanation that the arabinose abundant in large amounts in the studied fraction could limit the access of enzymes that modify the RG-I backbone.

### Functional groups

**Figure 3:**
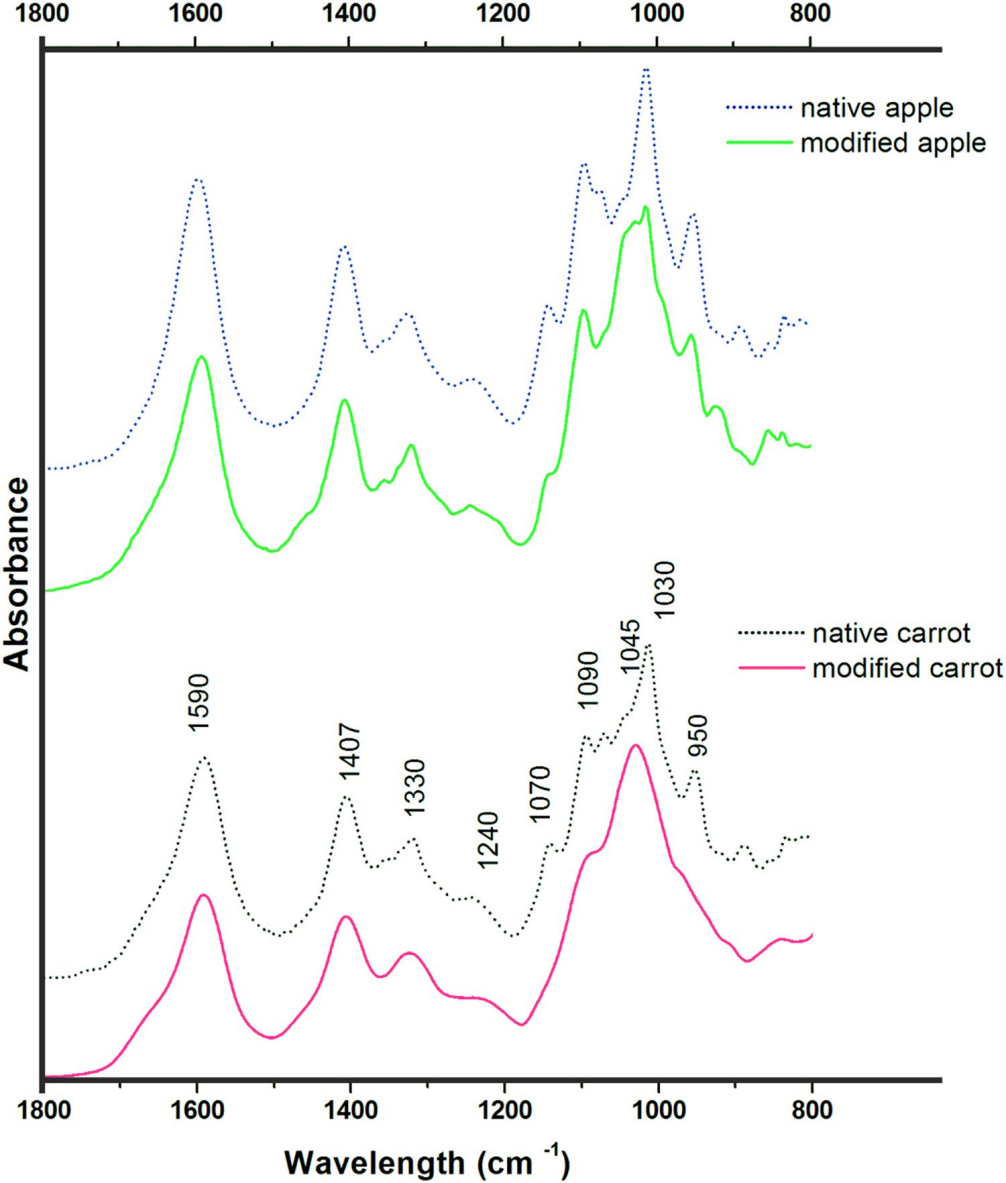
Attenuated total reflectance (ATR)–FTIR absorbance spectra of DASP extracted from apple and carrot in their native states (dotted lines) and after E3 enzymatic modification (solid lines) in the range of 1800–800 cm^−1^.

The FTIR spectra obtained for DASP-A and DASP-C, both native and treated with E3 for 120 min, are shown in Fig. 3. The overall shape of a polysaccharide spectrum is determined by the polysaccharide composition of the backbone but can also be strongly influenced by the side chain constituents^20^. The wavelength and intensity of the bands allow the evaluation of possible changes in polysaccharide composition. For all samples, characteristic absorption regions can be distinguished. The shape of each spectrum had a similar pattern, which is characteristic of DASP polysaccharides^2,21,22^.

For all samples, an absence of a band in the range of 1745–1700 cm^−1^, which is related to the vibration of esterified groups, suggests a lack of esters in the studied pectin fractions. This was quantified using HPLC measurements, and no methyl groups were detected for native pectin from either source. This was probably caused by the de-esterification of the pectin during sodium carbonate extraction^16^. The DA values determined by HPLC were 5.59% and 7.48% for native apple and carrot, respectively. This suggests incomplete degradation of the ester linkage between the acetyl group and the GalA residue in the pectin chains; however, these low values are supported by the lack of peaks on the FTIR spectra for acetylated carbonyl groups (1730 cm^−1^) and the stretching of C–O–C in acetyl ester (1250 cm^−1^), which is characteristic of acetylated pectic materials^23^. A broad band in the FTIR spectrum of the tested pectin, in the range of approximately 1260–1200 cm^−1^, could be considered the peak corresponding to C–O–C stretching; however, in this case it could also be caused by C–O stretching vibrations in pectin, as was previously described for DASP from pear^24^. Nevertheless, the lower intensity of the broad band around 1240 cm^−1^ for both samples treated with RGAE could be the effect of partial de-esterification of acetyl groups.

The band around 1590 cm^−1^ was assigned to the asymmetric stretching of COO– in polygalacturonic acid, representing non-esterified carboxyl groups in pectin, while the peak at approximately 1407 cm^−1^ represented symmetric stretching vibrations of carboxylic anions. The band at 1330 cm^−1^, which was assigned to ring vibration, was present for all samples. As previously shown, the DASP fraction showed a high intensity at this peak compared to the fractions extracted with water and imidazole^18^. Based on FTIR spectrum absorbencies in the range of 1200–900 cm^−1^ (fingerprint region), it is possible to determine groupings that are specific to each polysaccharide. The main component influencing the change in the shape of this fingerprint region due to enzymatic treatment is GalA, which shows main absorbance regions at approximately 1140, 1090, 1070, and 1030 cm^−1^. However, it has been shown that different pectic compounds also can show different characteristic positions of maximum bands in this region^21,25^. The absorbance bands at approximately 1075 cm^−1^ and 1045 cm^−1^ also suggest the presence of RG-I domains^20^. Changes in the intensity of bands in this region and/or the disappearance of peaks observed for modified fractions may suggest fragmentation changes in the DASP main chain. The peak at about 950 cm^−1^, which is characteristic of RG-I^21^, was assigned to galactose side chains^10^ and did not change in intensity for the E3 modified DASP-A. In contrast, its disappearance was observed for the enzymatically treated DASP-C. This could be the result of rhamnose removal and, therefore, galactan side chain loss for the carrot sample. Bands in the wavenumbers in the range of 900–800 cm^−1^ belong to the anomeric region and can be used to differentiate the α- and β-configurations of anomeric carbon. Peaks at approximately 890–850 cm^−1^ indicate the presence of galactopyranose and arabinofuranose units in a sample^20^. For both DASP-C and DASP-A, a disappearance of the peak at 890 cm^−1^ was observed for modified pectin. In addition, for DASP-A, a reduction in the peak intensity at 850 cm^−1^ was observed. These changes may suggest a rearrangement of the bonds related to side chains in the pectin molecule.

### Rheological properties

**Figure 4:**
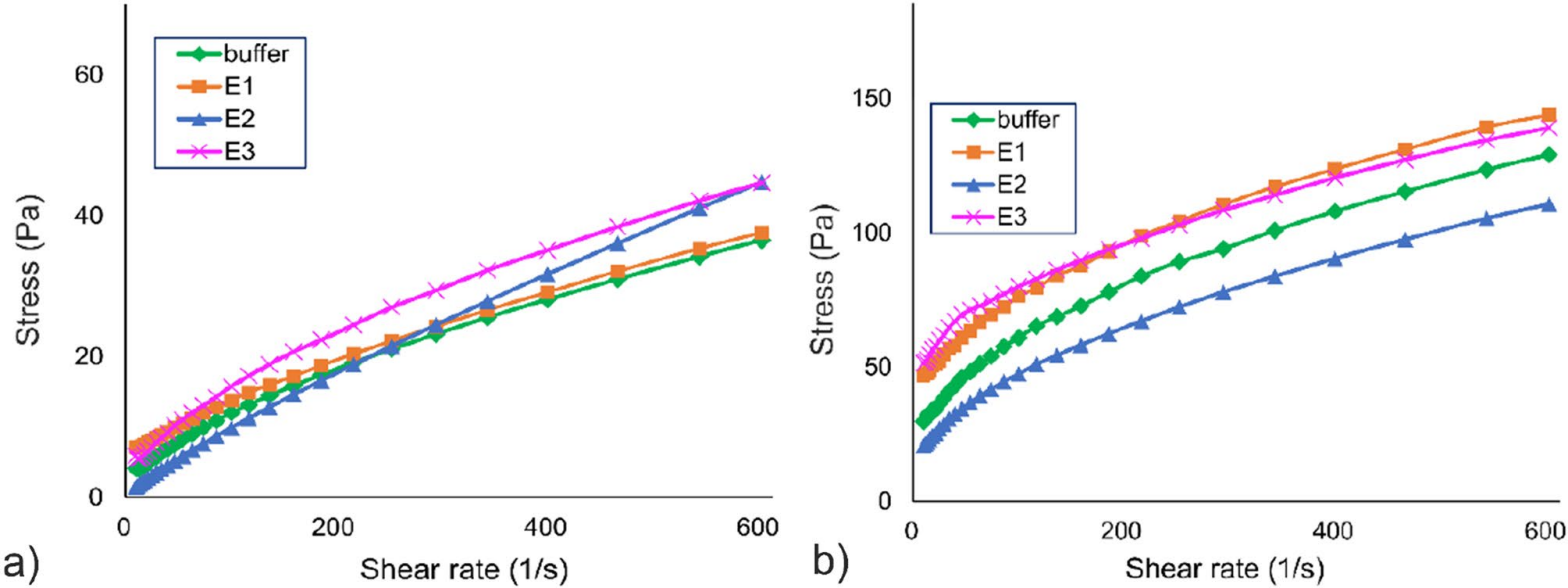
Averaged upward flow curves (shear stress vs. shear rate) for apple (**a**) and carrot (**b**) DASP solutions treated with different enzymatic combinations. E1: DASP water solution with RGAE and RGL; E2: DASP water solution with ABF; E3: DASP water solution with RGAE, RGL, and ABF.

Flow curves collected after 6% DASP-A and DASP-C solutions were treated with buffer or enzymes for 120 min are shown in Fig. 4. A power law (Ostwald–de Waele) and the Herschel–Bulkley fluid model were fitted to the shear rate–shear stress curves for all samples. The consistency coefficient (*K*) and flow behavior index (*n*) were used to describe fluid behavior (Table 1). All *n* values were less than 1, showing that samples behaved as pseudoplastic shear-thinning fluids, as reported previously for other pectin solutions^26^. This indicates that their apparent viscosity decreased with increasing shear rate and that the macromolecular network was oriented or deformed in the direction of flow.

**Table 1:** Rheological properties of native and modified DASP from apple and carrot. *G*′, elastic (storage) modulus;* G*″, viscous (loss) modulus; tan δ, loss factor; *K*, consistency coefficient; *n*, flow index; *G*_0_, initial shear stress.

Parameter	Unit	Pectin source							
		Apple				Carrot			
		BUFFER	E1 (RGAE + RGL)	E2 (ABF)	E3 (RGAE + RGL + ABF)	BUFFER	E1 (RGAE + RGL)	E2 (ABF)	E3 (RGAE + RGL + ABF)
G′	Pa	310.83 ± 231.30^ab^	1154.61 ± 139.91^a^	32.29 ± 17.23^ab^	338.77 ± 145.85^ab^	405.53 ± 28.92^b^	167.26 ± 154.87^ab^	43.67 ± 25.38^ab^	207.33 ± 119.49^ab^
G′/*G*″		7.52 ± 3.14^ab^	10.74 ± 1.42^b^	4.38 ± 0.99^ab^	8.34 ± 1.43^ab^	6.77 ± 0.51^ab^	6.44 ± 1.08^ab^	3.51 ± 0.93a	6.93 ± 2.48^ab^
tan δ		0.14 ± 0.00^abc^	0.09 ± 0.01^bc^	0.24 ± 0.05^a^	0.13 ± 0.03a^b^	0.15 ± 0.01^abc^	0.16 ± 0.03^abc^	0.30 ± 0.07^c^	0.15 ± 0.07^ab^
Flow point	%	6.07 ± 3.83^ab^	5.76 ± 1.26^bc^	16.09 ± 4.50a^b^	3.77 ± 1.95^a^	1.93 ± 0.85^a^	12.85 ± 7.76a^bc^	20.60 ± 10.64^c^	3.77 ± 1.62^ab^
Linear viscoelasticity limit	%	3.72 ± 1.83^abc^	1.79 ± 0.50^bc^	5.76 ± 2.20^a^	1.54 ± 0.75^a^	1.34 ± 0.90^a^	3.41 ± 1.47^abc^	7.20 ± 2.23^c^	1.88 ± 0.79^ab^
Power law model									
*K*	Pa s	2.59 ± 3.99^a^	1.39 ± 1.46^a^	0.19 ± 0.09^a^	2.62 ± 2.76^a^	9.33 ± 3.66^ab^	20.40 ± 13.57^b^	5.42 ± 2.16^a^	23.56 ± 12.54^b^
*n*		0.56 ± 0.16^bc^	0.63 ± 0.19^ cd^	0.86 ± 0.07^d^	0.54 ± 0.13^abc^	0.41 ± 0.06^abc^	0.32 ± 0.14^ab^	0.49 ± 0.09^abc^	0.29 ± 0.08^a^
Herschel–Bulkley model									
*G*_0_	Pa	3.63 ± 5.75^a^	3.21 ± 4.08^a^	0.06 ± 0.11^a^	3.71 ± 5.61^a^	10.79 ± 6.97^ab^	31.17 ± 20.41^bc^	7.94 ± 4.57^a^	32.98 ± 16.12^c^
*K*	Pa s	1.07 ± 1.16^abc^	0.46 ± 0.25^ab^	0.19 ± 0.09^a^	1.28 ± 0.9^abc^	4.76 ± 0.65^d^	3.07 ± 1.68^ cd^	2.79 ± 0.95^bcd^	5.08 ± 2.31^d^
*n*		0.63 ± 0.12^ab^	0.73 ± 0.09^bc^	0.87 ± 0.07^c^	0.61 ± 0.1^ab^	0.50 ± 0.03^a^	0.59 ± 0.14^ab^	0.58 ± 0.05^ab^	0.48 ± 0.04^a^
Viscosity (at 10 s^−1^)	Pa s	0.93 ± 0.89^ab^	0.65 ± 0.55^a^	0.14 ± 0.05^a^	1.58 ± 1.17^ab^	3.13 ± 0.87^bc^	4.91 ± 2.08^c^	2.13 ± 0.77a^b^	5.19 ± 1.90^c^
Intrinsic viscosity	mg L^−1^	158.33	148.53	151.56	123.29	293.37	242.31	160.28	147.51

Different letters indicate statistically significant differences (ANOVA, *p* < 0.05).

The E2 (ABF) treatment led to a decrease in *K* for both fractions, indicating a weakening of the binding of the network. It was concluded that arabinose side chains were involved in macromolecular entanglements in the native fractions, which resulted in higher viscosity for the pectin in buffer^27^. The strong impact of ABF on the structure of DASP could be caused by the relatively high content of arabinose in the tested fractions. The content of this monosaccharide was 23.6 ± 0.1 mol% in DASP-A and 19.8 ± 2.3 mol% in DASP-C (Table S1). Moreover, the tested fractions differed in rhamnose content: 3.8 ± 0.2 mol% for DASP-A and 8.4 ± 1.8 mol% for DASP-C. It is worth noting that, for DASP-A, which contains greater amounts of arabinose, decreases in viscosity and pseudoplastic character after incubation in ABF were much more pronounced. The suggested role of arabinose in the formation of a compact network was supported by a decrease in the yield stress (*G*_0_), which describes the minimum shear rate needed to initiate flow of the material, of samples treated with ABF. In contrast, a strong increase in this parameter, which was observed with the E3 treatment, indicated the formation of a dense network, which was resistant to mechanical disruption, for the debranched polymer. An increase in *K* with the E3 treatment, combined with a decrease in the flow index, suggests a stronger pseudoplastic character of the DASP solution after simultaneous deacetylation and removal of arabinose and rhamnose. The DASP-C sample, after modification with this enzymatic cocktail, showed the highest pseudoplasticity of all tested solutions. The E3 treatment also resulted in an increase in the viscosity of both sources. This may indicate a greater possibility of particle movement controlled by the entanglements of side chains attached to the rhamnose units as well as acetyl groups, which can hinder the adoption of binding-favorable conformations by the polymer^28^. In addition, rhamnose inclusions themselves can limit the cross-linking of chains^29^. An increase in the viscosity of DASP was previously observed during storage of carrot roots^26^. That study hypothesized that hydrogen bonding between smooth pectin chains and hydrophobic interactions by the methyl groups of pectin chains had occurred as a result of the enzymatic modification naturally occurring in roots. Therefore, it is suspected that E3 treatment resulted in predominantly unbranched acid polymers. Considering the low DM as a result of extraction with sodium carbonate, under these conditions the tendency to self-aggregate in deionized water is reduced. Hence, molecules adopt a more extended conformation that favors interactions between the chains^30,31^. This interpretation is supported by the recently highlighted role of intermolecular interactions in the mechanical properties of LM pectins, whose extended conformation at neutral pH can increase the elastic character of the mixture^32^.

To gain insight into the viscoelastic properties of the studied solutions, oscillatory measurements were performed. All the investigated samples exhibited behavior that was more solid-like than liquid-like, as evidenced by the storage modulus *G*′ being much greater than the loss modulus *G*″ in amplitude sweep tests. This was confirmed by values of the loss factor tan δ < 1 (*G*′ > *G*″).

A decrease in the storage modulus *G*′ was noted as a result of the E1 treatment for fractions from both sources. As a result of potential depolymerization of the RG-I backbone, this process reduces the average molecular weight of the polymer, leading to decreased entanglements and, hence, overall network strength reduction and disruption of crosslinking. Flow point and linear viscoelasticity values slightly decreased for DASP-A, which was a consequence of the process described; however, for DASP-C, the opposite trend was observed. The increase in these parameters suggests that the structure of deacetylated DASP-C, which is a result of enzymatic modification with RGAE, is more resistant to deformation of this material.

A large decrease in the storage modulus *G*′, which was observed after removing the arabinose side chains (E2 treatment), combined with an increase in the loss factor *G*″, indicated a significant decrease in the elastic properties of pectic gels. This may suggest that arabinose chains, as binding points in the pectin network, exhibit solid-like behavior. For RG-I–enriched pectin, arabinose was involved in gel formation under cation and acid conditions, and improved network formation and enzymatic debranching resulted in a decrease in side-chain entanglements and, hence, looser pectin molecule conformation^27^. Similarly, decreases in elastic properties and breaking force have been observed for debranched highly methylated citrus pectin gels^33^. This study also showed that untreated and debranched pectin gels were governed by the same type of interactions. However, for gels formed by less branched pectins, the network became less entangled, with fewer inter-chain connections, between the polymer molecules, which resulted in an overall decrease in elasticity. For the E2 treatment, increases in the flow point and linear viscoelasticity limit were observed, which indicates that the system was able to retain the molecular properties of the pectin network as the strain increased. It is worth noting that the E2 treatment, by selectively excising the arabinose units, left the galactan side chains intact. Therefore, it is possible that aggregate-stabilizing properties of galactans became apparent in this sample, as was shown by another study^34^. The rheological parameters obtained may indicate that, for pectin at a concentration of 6%, molecular association occurred with the formation of intermolecular interactions. When the E3 enzyme combination was applied, similar viscoelastic parameters were obtained for both sources. This may indicate that the degradation of arabinose side chains and deacetylation and removal of rhamnose chains have major effects on the observed differences between the DASP of these two materials.

As a result of enzymatic modification, only a small effect on intrinsic viscosity was observed for DASP-A (E3 had the greatest impact on this source). A much more significant effect of enzymatic treatment on intrinsic viscosity was noted in the case of DASP-C. The observed decrease in intrinsic viscosity following enzymatic modification suggests a significant impact on the molecular structure, particularly in DASP-C. This reduction in intrinsic viscosity, which was notable after the E2 and E3 treatments, indicated the formation of compact molecular aggregates, as shown in AFM images (Fig. 1g,h). These aggregates may signify an increase in molecular flexibility and compactness, possibly resulting from the dissociation of supramolecular aggregates in the native fraction and the reduction of intramolecular forces^30^ induced by selective degradation of the pectin chain. The different reactions of the apple and carrot fractions to enzymatic modification may be related to differences in their monosaccharide composition of initial fractions (Table S1). Since solutions lacked sucrose and cations, apart from the low salt content of the enzyme buffer, the network formed in the solution was the result of the DASP fraction’s natural tendency to self-assemble, which was previously noted and observed with AFM at low concentrations^10^. Therefore, it can be expected that the association of pectin chains of the DASP fraction in aqueous solution in the absence of cations occurs by two mechanisms. For native DASP polymers, which are composed of both smooth and hairy structures, neutral side chains are involved in providing multiple short-range attachment points for intermolecular entanglement, which is more favorable than electrostatic repulsion between GalA chains. In contrast, for smooth and linear regions, which are rich in GalA at neutral pH, strongly charged molecules cause intramolecular repulsion; thus, a more extended conformation results^8^. Short linear sections with high mobility cause interactions between the chains to be more favorable than self-aggregation.

## Conclusions

This study demonstrated changes in the structure and rheological properties of DASP fractions extracted from apples and carrots under the influence of enzymes that selectively modified the pectin backbone and side chains. The structure of pectin in the DASP fractions had a significant influence on rheological properties. This was supported by changes in the structure, chemical composition, and rheological properties of samples observed as a result of enzymatic modification of RG-I fragments. The removal of rhamnose units, simultaneously with the deacetylation and removal of arabinose side chains, resulted in similar rheological parameters of pectic gels from two plant sources that were different from the properties of the unprocessed samples. Therefore, it can be concluded that rhamnose may be the factor determining the properties of pectin matrices in solution. Modification with ABF had the greatest impact on the properties of this pectin fraction. Arabinose, which is present in side chains, is involved in the formation of the pectin network and affects the pseudoplastic properties and viscosity, but not the mechanical strength, of pectin solutions. At the same time, a significant increase in the lengths of the chains after the removal of arabinose indicates that the side chains are a hindrance limiting the binding of polymer chains.

It can thus be concluded that the association of the pectin chains of DASP fractions in an aqueous solution in the absence of cations may occur due to the crucial role of intermolecular interactions according to two mechanisms: side chains as short-range attachment points and an extended linear HG conformation favoring inter-chain interactions over self-association.

## Materials and methods

### Pectin source

The research material included apples cv. Najdared (*Malus domestica* Borkh*.*) and carrots cv. Brava *(Daucus carota* subsp. *sativus*). Material was harvested in Poland in October 2020 and then stored in a cold room at 2 °C and normal atmosphere for 2 days until preparation. Pulp was prepared from 102 kg of raw apples and 34 kg of raw carrots. Both were peeled and sliced. The juice was pressed, and the remaining pomace was homogenized. Then, the prepared material was frozen at − 18 °C for further analysis. Alcohol-insoluble residue (AIR) was prepared identically for both plants, according to the method described by Renard^35^ with some modifications. The pulp was mixed with ~ 70% ethanol (solid–liquid ratio of 1:10, w/v) for 15–30 min, and then the mixture was filtered on a nylon filter, and the residue was stirred again with ethanol. This procedure was repeated until a negative result of the phenol–sulfuric acid test^36^ was obtained, thereby confirming the absence of sugar in the pulp. Next, the sample was washed with 96% ethanol and subsequently with acetone and then dried at 45 °C.

### DASP extraction

Sequential extraction was performed for both sources according to the method proposed by Redgwell and Selvendran^37^ with certain modifications. AIR was stirred in deionized water (solid–liquid ratio of 1:9, w/v) for 24 h at 21 °C and then centrifuged (5000 rpm). Supernatant was collected as a water-soluble pectin fraction, and the sediment was mixed with 0.1 M cyclohexane-trans-1.2-diamine tetra-acetate (CDTA, pH 6.5) and stirred at 21 °C for 24 h. After centrifugation, the supernatant was separated as a chelate-soluble pectin fraction, and 0.05 M sodium carbonate (Na_2_CO_3_), with the addition of 20 mM sodium borohydride (NaBH_4_), was added to the residue and stirred for 24 h at 21 °C. The DASP fraction was collected after centrifugation as a supernatant and encoded as DASP-A or DASP-C, for apple-extracted or carrot-extracted samples, respectively. The DASP fraction was dialyzed in an open system using ZelluTrans/ROTH® membranes (Carl Roth GmbH & Co. KG, Germany; MWCO 3500 Da), and then crude extract was lyophilized. DASP from apple and carrot was extracted with a yield of 0.44% and 0.61% of fresh weight (25.58 and 21.30% of dry weight), respectively^13^. The chemical composition of DASP from both sources is presented in Table S1 (Supplementary Information).

### Determination of DA and DM

To determine the DM and DA of pectin, samples were saponified with 0.2 M NaOH to produce methanol and acetic acid, which were then measured by HPLC (C18 column, Bionacom velocity LPH-C18, 300 Å, 4.6 × 250 mm, 5 microns, RI detector). The method of Levigne et al.^38^ with some modifications by Yu et al.^39^ was used. Pectin samples of 5 mg were suspended in 0.5 mL 0.2 M NaOH and incubated at 4 °C for 120 min. Then, the mixture was neutralized with 0.5 mL 0.2 M H_2_SO_4_, centrifuged for 10 min, filtered through a 0.22 µm syringe filter, and injected into the HPLC column (injection volume 20 µL, mobile phase 4 mM sulfuric acid at a flow rate of 0.8 mL min^−1^). Standard solutions of methanol and acetic acid were prepared and analyzed under the same conditions. The analysis was performed in triplicate.

### Enzymatic treatment

The DASP fractions from apples and carrots were treated with enzymes that degrade the RG-I backbone and its side chains. Three types of enzymes were used: RGAE (BtRme NC (CE NC), BT4158, E.C. 3.1.1), RGL (BtRge9A (PL9), BT4183, E.C. 4.2.2.23), and ABF (CjAbf51B (GH51), E.C. 3.2.1.55). All enzymes were purchased from NZYTech and provided in 35 mM Na–HEPES buffer (pH 7.5, 750 mM NaCl, 200 mM imidazole, 3.5 mM CaCl_2_, and 25% v/v glycerol).

The quantities of enzymes were selected on the basis of their activity^15,40,41^ and the chemical composition of DASP with 10% excess. Per 1 mg of DASP, 0.22 U of RGL, 9.9 U of RGAE, and 2.1 U of ABF were used. Volumes were obtained according to protein concentrations (RGAE: 0.5 mg mL^−1^; RGL: 0.5 mg mL^−1^; ABF: 0.25 mg mL^−1^).

DASP water solutions were incubated in three different enzyme cocktails (Table 2). As a control (no enzymatic treatment), the same buffer solution in which the enzymes were delivered was added to DASP water solutions.

**Table 2:** Code list and description of the treatments used.

Treatment code	Description
E1	RGAE (9.9 U mg^−1^) + RGL (0.22 U mg^−1^)
E2	ABF (2.1 U mg^−1^)
E3	RGAE (9.9 U mg^−1^) + RGL (0.22 U mg^−1^) + ABF (2.1 U mg^−1^)
BUFFER	35 mM Na–HEPES buffer, pH 7.5, 750 mM NaCl, 200 mM imidazole, 3.5 mM CaCl_2_, and 25% (v/v) glycerol

The pH values of all tested solutions were in the range of 7.30–7.60. Control and enzyme-treated samples were incubated in a water bath at 37 °C for 120 min. After incubation, the samples were cooled in an ice bath for 5 min to stop the enzymatic reactions, mixed, and used for further analysis.

### AFM imaging and analysis

After enzymatic modification, 30 μL of each DASP sample, with a concentration of 0.02 mg mL^−1^, was distributed on freshly cleaved mica sheets (EMS, Hatfield, PA, USA) using a POLOS SPIN150i-NPP spin coater (SPS-Europe B.V., Putten, the Netherlands). Samples were observed (after drying in a desiccator at 22 °C overnight) using a Multimode 8 AFM with a Nanoscope V controller (Bruker, Billerica, MA, USA), a SCANASYST-AIR-HR cantilever (Bruker, Billerica, MA, USA), and a nominal spring constant of 0.4 N m^−1^. The observations were conducted in ambient air at room temperature. The following scan settings were applied: scan size of 4 × 4 μm with a resolution of 1024 × 1024 points and a scan rate of 3.91 Hz. In total, at least 10 images of each sample representing different regions of the mica sheets were collected. Preliminary processing of images was conducted using Gwyddion 2.52 ^42^. Geometrical features of DASP structure were calculated with a MATLAB R2011a script (MathWorks, Natick, MA, USA). Molecules visible on the AFM images were classified as hairy or smooth, according to the presence of branch points in the chains (Fig. 1). A single segment was defined as the section between the nearest branching points or between a branching point and the end of the molecule. The total length of hairy molecules (as the sum of their segments), the length of smooth molecules, and the length of a single segment were determined. Moreover, the average number of branches (segments) per molecule was determined.

### FTIR spectroscopy

DASP was dissolved in deionized water at a concentration of 6% (m/v). After preparation, samples were vortexed (3000 rpm) and then mixed overnight. DASP was treated with mixture of E1, E2, or E3 and incubated as described the “Enzymatic treatment” section. Subsequently, samples were cooled in ice for 5 min, mixed overnight, and freeze-dried. FTIR spectra were collected using a Nicolet 6700 FTIR (Thermo Scientific, Madison, WI, USA) with the Smart iTR attenuated total reflectance (ATR) sampling accessory. All samples from both sources were analyzed under the same conditions. Spectra were collected in the range 4000–650 cm^−1^ with a spectral resolution of 4 cm^−1^. Measurements were performed in three repetitions with 200 scans averaged for each repetition. The baseline corrections were performed using OMNIC software (Thermo Scientific). The final average spectrum was calculated from collected data and normalized to 1.0 at 1019 cm^−1^ using OriginPro 8.5 software (OriginLab Corporation, Northampton, MA, USA).

### Determination of rheological properties

DASP 6% (m/v) solutions were prepared in the same way as for FTIR analysis (“FTIR spectroscopy” section); treated with a mixture of E1, E2, or E3; and incubated as described in the “Enzymatic treatment” section. Subsequently, samples were cooled in ice for 5 min. After cooling, samples were stabilized at room temperature before the oscillatory test and flow behavior measurements were performed.

For intrinsic viscosity measurements, a stock solution of 20 mg mL^−1^ DASP in deionized water was prepared. A mixture of enzymes (E1, E2, or E3) was added to the well-dissolved sample. After 120 min of incubation, the sample was cooled in ice. From the stock solution, dilutions (5–18 mg mL^−1^) were prepared to obtain intrinsic viscosity curves.

Rheological measurements were performed at 20 °C using a Discovery Hybrid Rheometer (HR-1) by TA Instruments (New Castle, PA, USA) with a cone plate sensor (40 mm diameter and 2.007° angle) with a 0.56 mm gap between the cone apex and the plate.

#### Viscoelastic properties

The oscillatory test was conducted with amplitude sweeps to describe the storage (G′) and loss (G″) moduli of the obtained networks. Measurements were performed while keeping the frequency at a constant value of 0.5 Hz and using a logarithmic sweep with strain in the range of 0.1–50% (25 points per decade; volume of the deposited sample 1 mL).

#### Flow behavior

Shear stress vs. shear rate dependences (flow curves) were measured between shear rates of 10–600 s^−1^ and 600–10 s^−1^ (logarithmic sweep, 15 points per decade). The viscosity was recorded at constant shear rate of 10 s^−1^. A power law model (Ostwald–de Waele model) and the Herschel–Bulkley model were applied to obtain flow curves in order to determine the rheological behavior of samples.

The power law model is given as Eq. (1):

1 $$\sigma = K\gamma^{n}$$

where $\sigma$ = shear stress, *K* = consistency index (Pa s^*n*^), γ = shear rate (s^−1^), and *n* = flow behavior index.

The Herschel–Bulkley model is described by Eq. (2):

2 $$\sigma = \sigma_{0} + K\gamma^{n}$$

where σ = shear stress, σ_0_ = initial shear stress (Pa), *K* = consistency index (Pa s^*n*^), γ = shear rate (s^−1^), and *n* = flow behavior index.

#### Intrinsic viscosity

The variable shear rate of 10–400 s^−1^ and 400–10 s^−1^ (logarithmic sweep; 25 points per decade) was used to measure viscosity of the solutions with a volume of the deposited sample of 0.80 mL. The intrinsic viscosities of samples were determined via an extrapolation of Eq. (3) to a concentration *c* equal to 0^43^:

3 $$\frac{{\eta_{sp} }}{c} = \left[ \eta \right] + k_{H} \left[ \eta \right]^{2} c$$

where η_sp_ = the specific viscosity that can be obtained from the relative viscosity (η _solution_/η_solvent_), *c* = polymer solution concentration, and *k*_H_ = the Huggins constant.

### Statistical analysis

The data were analyzed with multi-way ANOVA and a post hoc Tukey honestly significant difference test (significance level *p* < 0.05) using the “stats” package (version 4.1.2) of R (R Core Team, 2013) and Statistica 13.1 (StatSoft, Krakow, Poland).

### Ethical approval

All methods were in accordance with the International Union for Conservation of Nature Policy Statement on Research Involving Species at Risk of Extinction and the Convention on International Trade in Endangered Species of Wild Fauna and Flora. The botanical material was harvested from a commercial orchard located in Ostrowiec (52°9′59.60′′ N, 20° 3′ 23.84′′ E) and an agricultural experiment station located in Dębowa Góra (51°51′8.38′′ N, 20°7′1.76′′ E), Poland.

### Supplementary Information

Supplementary Information.

## Data Availability

The datasets generated during and/or analyzed during the current study are available from the corresponding author on reasonable request.
