# Supplementary material for: Effect of enzymatic modification on the structure and rheological properties of diluted alkali-soluble pectin fraction rich in RG-I
Source: Sci Rep. 2024 May 20;14:11454. doi: 10.1038/s41598-024-62180-2 (PMC11106296; doi:10.1038/s41598-024-62180-2)
Supplement: Supplementary file 1 — Supplementary Information. [file 41598_2024_62180_MOESM1_ESM.docx]

**Supplementary Information**

**Effect of enzymatic modification on the structure and rheological properties of diluted alkali-soluble pectin fraction rich in RG-I**

**Adrianna Kaczmarska, Piotr M. Pieczywek, Justyna Cybulska, Artur Zdunek***

Institute of Agrophysics, Polish Academy of Sciences, Doświadczalna 4, 20-270 Lublin

*Corresponding author: Artur Zdunek (a.zdunek@ipan.lublin.pl, phone: +48 81 744 50)

1. **Supplementary Materials and Methods**

**Monosaccharide composition**

Monosaccharides were determined as 1-phenyl-3- methyl-5-pyrazolone (PMP) derivatives according to the method developed by Lv et al. (2009) and Zhang et al. (2018) with some modifications. Lyophilized pectin fractions were treated with 2 M methanolic HCl at 80 ºC for 18 h, and then with 2 ml of 3 M trifluoroacetic acid (TFA) solution at 100 °C for 3 h. Then derivatization of the resulted monosaccharides and uronic acids with PMP was carried out by adding 1 ml of water and 50 µL of 0.3 M NaOH, mixing and finally adding 50µl of a 0.5 M solution of PMP in methanol. Samples were incubated at 70 °C for 60 min, cooled, neutralized with 50 µl of 0.3 M HCl and extracted thrice with chloroform. Before injection into the HPLC system, each sample was filtered through a 22-µm membrane. Standards of the monosaccharides and uronic acids (arabinose, fucose, galactose, galacturonic acid, glucose, glucuronic acid, mannose, rhamnose, and xylose) were treated in the same way as pectin samples.

The PMP-labelled samples were analysed using a Young Lin 9300 HPLC system with UV/VIS detector (Young Lin Bldg, Anyang, Korea) equipped with a Zorbax Eclipse XDB-C18 (4.6 mm i.d. × 250 mm, 5 µm) analytical column coupled with an Agilent Eclipse XDB-C18 guard column (4.6 mm i.d. × 12.5, 5 μm). The mobile phase was composed of A: 0.1 M phosphate buffer (pH 6.7) and B: 50 % v/v solution of 0.1 M phosphate buffer in acetonitrile, at a ratio A:B of 69:31 % (v/v) under isocratic elution mode. The injection volume was 20 μl, with a flow rate of 1.8 ml∙min-1 at 30 ºC and the detection wavelength was 246 nm. Analysis was performed in three independent repetitions.

1. **Chemical composition of samples**

Table S1. Monosaccharides and uronic acids composition of generated DASP samples from apple and carrot. Man, mannose; Rha, rhamnose; GlcA, glucuronic acid; GalA, galacturonic acid; Glc, glucose; Gal, galactose; Xyl, xylose; Ara, arabinose; Fuc, fucose; nd, not detected.

| Pectin source | Treatment | Man | Rha | GlcA | GalA | Glc | Gal | Xyl | Ara | Fuc |
| --- | --- | --- | --- | --- | --- | --- | --- | --- | --- | --- |
|  |  | mol% | | | | | | | | |
| Apple | BUFFER | 1.6±0.2 | 3.3±0.1 | 0.3±0.6 | 56.1±0.6 | 0.3±0.1 | 12.8±0.7 | 1.8±0.2 | 23.6±0.1 | 0.3±0.1 |
|  | E1 | 1.7±0.2 | 3.6±0.3 | 0.3±0.1 | 57.5±3.2 | 0.4±0.1 | 12.4±1.1 | 1.4±0.1 | 22.4±2.6 | 0.2±0.1 |
|  | E2 | 1.6±0.1 | 3.4±0.5 | 0.3±0.2 | 55.4±0.9 | 0.5±0.2 | 12.3±1.0 | 1.1±0.3 | 25.3±1.1 | 0.1±0.0 |
|  | E3 | 1.2±0.3 | 3.5±0.7 | 0.7±0.1 | 56.3±2.2 | 0.6±0.2 | 11.7±1.3 | 1.5±0.3 | 24.3±1.4 | 0.2±0.1 |
| Carrot | BUFFER | 0.6±0.2 | 7.0±0.7 | 0.2±0.1 | 44.8±2.9 | 0.2±0.1 | 27.2±0.3 | nd | 19.8±2.3 | 0.2±0.1 |
|  | E1 | 0.7±0.1 | 8.2±1.1 | 0.1±0.1 | 44.4±1.5 | 0.2±0.1 | 26.7±1.0 | nd | 19.4±0.5 | 0.3±0.1 |
|  | E2 | 0.9±0.0 | 7.4±2.1 | 0.3±0.1 | 45.1±1.2 | 0.5±0.2 | 24.8±1.1 | nd | 20.7±2.1 | 0.4±0.2 |
|  | E3 | 0.8±0.2 | 7.1±1.6 | 0.2±0.1 | 45.0±1.2 | 0.2±0.1 | 29.9±1.4 | nd | 16.8±1.9 | 0.2±0.1 |
